# Basic ICT adoption and use by general practitioners: an analysis of primary care systems in 31 European countries

**DOI:** 10.1186/s12911-015-0185-z

**Published:** 2015-08-22

**Authors:** Sabina De Rosis, Chiara Seghieri

**Affiliations:** Scuola Superiore Sant’Anna, Institute of Management, Laboratorio Management e Sanità, piazza Martiti della Libertà 33, Pisa, 56127 Italy

**Keywords:** Primary care, General practitioners, ICT, Computer, E-health, Europe

## Abstract

**Background:**

There is general consensus that appropriate development and use of information and communication technologies (ICT) are crucial in the delivery of effective primary care (PC). Several countries are defining policies to support and promote a structural change of the health care system through the introduction of ICT. This study analyses the state of development of basic ICT in PC systems of 31 European countries with the aim to describe the extent of, and main purposes for, computer use by General Practitioners (GPs) across Europe. Additionally, trends over time have been analysed.

**Methods:**

Descriptive statistical analysis was performed on data from the QUALICOPC (Quality and Costs of Primary Care in Europe) survey, to describe the geographic differences in the general use of computer, and in specific computerized clinical functions for different health-related purposes such as prescribing, medication checking, generating health records and research for medical information on the Internet.

**Results:**

While all the countries have achieved a near-universal adoption of a computer in their primary care practices, with only a few countries near or under the boundary of 90 %, the computerisation of primary care clinical functions presents a wide variability of adoption within and among countries and, in several cases (such as in the southern and central-eastern Europe), a large room for improvement.

**Conclusions:**

At European level, more efforts could be done to support southern and central-eastern Europe in closing the gap in adoption and use of ICT in PC. In particular, more attention seems to be need on the current usages of the computer in PC, by focusing policies and actions on the improvement of the appropriate usages that can impact on quality and costs of PC and can facilitate an interconnected health care system. However, policies and investments seem necessary but not sufficient to achieve these goals. Organizational, behavioural and also networking aspects should be taken in consideration.

## Background

Among the drivers of change in the health care sector, the advent of the computer and of its applications have revolutionised health care systems and services [1, 2]. The adoption and use of information and communication technologies (ICT) are increasingly seen as support, redesign and improvement tools for health care delivery, especially in primary care (PC). ICT can contribute to address the main challenges of cost reduction and improvements in equity and quality of care [3–6]. In fact, there is growing scientific evidence on the potential of ICT adoption and use in PC, regarding appropriate services [7–9] and high-quality and efficient care [7, 10–13].

Health information technologies, such as electronic health records (EHRs) and digital interactions, have been demonstrated to contribute to a more effective delivery of PC services [14–22]. In particular, they can (i) facilitate adherence to guidelines in clinical practice and reduce medical errors with better knowledge management and evidence-based decision making [23–25]; (ii) contribute to integration and continuity of care with improved communication among physicians, patients and specialists [26]; and (iii) produce savings of time and costs [24, 27, 28]. Moreover, the health care workforce is expected to experience improvements in efficiency, delegation support and remote care if health ICT and related electronic applications are adopted in integrated and comprehensive ICT systems [29].

ICT solutions are increasingly available to PC physicians without great investments in hardware, software and maintenance. The development and availability of technologies for health care have facilitated the introduction of policies in several countries, aimed at increasing their rapid adoption [13, 30, 31]. However, each country might respond differently to the actual challenges with various approaches towards potential solutions and drivers of growth, including the technological equipment for primary health care. In addition, the level of social and economic development, the characteristics of health care systems and the level of managerial capacity could make some technologies suitable for certain countries but not others [32]. In recent decades, a number of countries have experienced the implementation of various ICT applications in PC, including follow-up consultations via email, online access to laboratory results, mobile access to radiology images and new communication tools [13]. However, a larger number of countries remain in the first stages of technological innovation in PC. These first stages relate to the development of information systems and the computerisation of PC functions, primarily identifiable in a shift from a paper-based processing/storage system to a computer-based one.

The specific features of each country can contribute to understanding the different states of ICT implementation and its applications in PC systems. We found evidence in the literature of an association between the size of PC practice (PCP) and the use of ICT. For example, a higher use of ICT is observed in larger practices than in single-GP practices [33, 34]. Furthermore, the inter-professional setting of a PCP can promote the shared adoption and use of technologies [35–38]. The location of the practice can also be a lever to the adoption and use of ICT. For instance, in rural areas, ICT can diminish the quality and equity disparities between urban and rural settings of health care [13, 39–41]. However, there is some evidence of the existence of a digital divide between metropolitan and rural areas, where rural zones present lower rates of general practitioners (GPs) using ICT [34, 42]. The presence of financial incentives in the specific remuneration model of the country can play a role in the deployment and use of ICT in health care. For example, in the USA, physicians salaried by capitated or budgeted organisations have been among the earliest adopters of EHRs and other comprehensive health IT [43, 44].

### Aims

To the best of our knowledge, most published studies regarding ICT in PC have been largely conducted in English-speaking countries or in Northern Europe [45–48]. The studies of the Commonwealth Fund on profiles of health care systems also concerned topics related to e-health including national policies and EHR-system implementation [20, 49, 50].

The European Commission promoted two studies on the adoption of e-health by European GPs: the first survey was conducted in 2007 [30] and the second in 2013 [51]. The 2013 survey aimed at measuring the adoption of e-health applications among GPs in the EU-27, Croatia, Iceland, Norway and Turkey, and used four key indicators: EHR; health information exchange, tele-health and personal health records. The study also aimed to explain the drives or barriers of adoption and to measure variations between 2007 and 2013. In 2010, a European study presented an analysis of the national policies of Member States on e-health, understood as ICT for health [31].

Given these premises, the overall aim of this study is to measure the current situation regarding the introduction and level of use of basic ICT in the PC systems of 31 European countries using data from the Quality and Costs of Primary Care in Europe (QUALICOPC) study [52]. Additionally, the study intends to measure variations in the period 1993–2012 using data from the European Study on GP Task Profiles (TP study) [53].

In particular, starting from the QUALICOPC data, our study focuses on ICT use by GPs because of their roles as the main gatekeepers for PC services [53]. Several computerised clinical functions were also investigated. In particular, we investigated “appropriate” applications of ICT in PC, which must be effective, cheap and safe. A basic appropriate ICT infrastructure in GP practice (the “tangible” aspects) consists of one or more available computers and an Internet connection [32]. Furthermore, appropriate ICT equipment is the basis for different e-health uses and computerised clinical functions (“intangible” aspects), which can be appropriate if they contribute to an improvement of the PC service delivery. (Note that e-health is defined as the delivery or improvement of information and health services through the Internet and related technologies [54].) The level of adoption of computers in PCP across Europe and the professional use of these technologies by GPs were analysed using selected questions from the QUALICOPC questionnaire to GPs. In particular, starting from existing studies on the appropriateness of computer uses [32, 55–57], we analysed the uses that: contribute to an improvement of PC delivery [10]; can be economically sustained with local resources; are acceptable to users and recipients; respond to local health needs (not of a small minority); are scientifically validated; and can be a part of a broader interconnected system and be integrated in the usual practice of GPs [58–60]. The ICT applications from QUALICOPC data that present the characteristics listed above are as follows: (i) record keeping; (ii) drug prescriptions; (iii) storage of tests results; (v) communication with other parties; and (vi) searching medical information [10].

Finally, we discuss our results to better understand the variations across Europe. The large amount of QUALICOPC data on different PC domains enabled us to analyse our results on ICT use with possible explanatory variables. In particular, we used data related to the characteristics of both GPs and PCPs. We used the following GP variables: age, gender, typology of employment (self-employment vs. employment), typology of remuneration (prospective, retrospective, other) and incentives. The salient PCP variables are as follows: patient population size and demographic characteristics (i.e., number of elderly), practice size (alone vs. shared) and location (urban, sub-urban, rural). We also discuss our results in light of variables at the country level, such as health system typology, expenditure on health care and health ICT and e-health strategies.

## Methods

The QUALICOPC project was funded by the European Commission under the Seventh Framework Program. It collected survey data from GPs and their patients in 31 European countries (EU-27 excluding France, FYR Macedonia, Iceland, Norway, Switzerland and Turkey) and three non-European countries (Australia, Canada [Ontario] and New Zealand) [52]. For the aim of our study, we only used data from the European countries.

In the QUALICOPC project, a consortium of six European partner institutes, coordinated by the Netherlands Institute for Health Services Research (NIVEL), developed and tested a number of hypotheses by concentrating on different domains: structure of care, quality of service provision, patients’ perceived quality of care, costs, equity, avoidable hospitalisation and good practices [52]. Scuola Superiore Sant’Anna (SSSA) was the Italian partner of the QUALICOPC project and was the leader of the work package on the costs and efficiency of PC systems. SSSA also participated in the work package on the structure of PC systems, analysing their ICT structure and use. The concept of structure of care refers to the characteristics of PC such as equipment and human resources and was also analysed in terms of ICT infrastructure and use. This study is the first analysis of basic ICT structure and use in PC using QUALICOPC data.

The QUALICOPC survey was conducted between 2011 and 2013, depending on the country (hereafter we will refer to the data as 2012 data). Three English-language questionnaires (one for GPs and two for patients) were developed by the QUALICOPC consortium after a previous study on other validated questionnaires [52]. The questionnaires were translated and piloted in three European countries (Belgium, the Netherlands and Slovenia) to a small sample of GPs and patients before finalising the source version for the European study. The pilot showed that the questionnaires were of acceptable clarity and applicability. The pilot phase also revealed the need for a further reduction of the questionnaire and the reformulation of several questions. Based on the findings of the pilot, the questionnaires were shortened and rephrased where needed. In this phase, the aim was to improve the intelligibility of questions without affecting their validity (because of a change in wording) [52]. In every participating country, an institute coordinated the data collection. The national coordinators were also responsible for the translation of the questionnaires. A team of PC experts in each country created draft translations that were verified by professional translators using a forward–backward methodology. The national coordinators were also responsible for organising, recruiting and collecting survey data from a representative sample of GPs in each country. A number of PC physicians were sampled in each country with the following targets: 80 GPs from small countries (Cyprus, Iceland, Luxembourg and Malta); 220 GPs from other countries. Where national registers of GPs were available, random sampling was used to select GPs from such lists (this method was used in most countries). In countries where no national registers exist, other sampling procedures were used, still providing a good representation the national situation (Finland, Greece, Hungary, Ireland, Italy and Norway).

Only one GP per practice or health centre was eligible to participate [52]. In most countries, the selected GPs were contacted via a mix of approaches (combinations of letters, email and telephone contact). Furthermore, it was common that one or more reminders were sent to the selected GPs.

Ethical approval was obtained in each country where needed, in accordance with national requirements (Table 1). Patients and GPs were informed about the study and had to provide their consent before filling out the questionnaires. Depending on the national requirements, written or oral informed consent was requested. The general procedure was that GPs were invited via letter, e-mail or telephone and gave their consent to participate in the study. Patients were invited by the fieldworker or practice staff to complete a questionnaire. All participants were informed about the study and participation was voluntary. Both patient and GP surveys were carried out anonymously. An identification number linked GP responses to the responses of their patients.

**Table 1:** List of the ethics committees that approved the QUALICOPC study in each country

Country	Ethics committee
Austria	Ethics committee of the Medical University of Vienna.
Belgium	University Hospital Ghent - Commission for Medical Ethics.
Bulgaria	The coordinator sent an official letter to the Ministry of Health which gave consent and support for the survey. The coordinator confirmed that there is no statutory requirement for ethical approval for this study.
Cyprus	National Bioethical Committee of Cyprus.
Czech Republic	General University Hospital linked to the First Faculty of Medicine, Charles University in Prague.
Denmark	The coordinator confirmed that there is no statutory requirement for ethical approval for this study.
Estonia	The national coordinator consulted with the Ethics Review Committee on Human Research of the University of Tartu. It was confirmed that there is no statutory requirement for ethical approval for this study.
Finland	The ethical committee of Pirkanmaa Hospital District.
Germany	Ethics Commision of the “Landesärtzenkammer Hessen”.
Greece	Bioethical committees of seventy hospital.
Hungary	National Ethical Committee.
Iceland	The Icelandic Bioethics Committee. A national committee under the Ministry of Welfare.
Ireland	Irish College of General Practitioners Research Ethics Committee – National Committee.
Italy	At Local Health Authorities level. Approval was requested from LHA Ethical Committees.
Latvia	Latvian Physicians Association Board of Certification
Lithuania	Kauno Regionus Biomedicininu Tyrimu Etikos Komitetas.
Luxembourg	National committee of Research Ethic (CNER) in Luxembourg.
Malta	University of Malta Research Ethics Committee.
Netherlands	The ethics committee of VU Medisch Centrum confirmed via an official letter that the research is outside the scope of the WMO Act (Medical Research Involving Human Subjects Act).
Norway	The coordinator confirmed that there is no statutory requirement for ethical approval for this study.
Poland	Bioethics approval of Jagiellonian University.
Portugal	Ethical committee of Lisbon and Oporto regions; the National Commission for Health Data Safety.
Romania	Scientific Committee of CPSS.
Spain	Research Units of Primary Care of the Autonomous Community in the Basque Country. In all other Atonomous Communities the study was approved at the Healthcare Area level.
Slovakia	The national coordinator consulted with the Council of the Slovak Society of General Practice. It was confirmed that there is no statutory requirement for ethical approval for this study.
Slovenia	National medical ethics committee.
Sweden	Regional Research Ethics Committte.
Switzerland	Ethical Committee of the University of Lausanne.
Turkey	Ethical committee of Kartal Research and Education Hospital in Istanbul.
United Kingdom	University of Lincoln School of Health and Social Care Ethics Committee; National Research Ethics Service.

In most countries, paper questionnaires were sent by mail to GPs and returned by mail to the national coordinator and then electronically read into the European database by a professional data management company. In other countries, GP questionnaires were filled out electronically online or via a tablet computer.

In the QUALICOPC survey for GPs, the use of computers in the PCP and the main purposes of computer use were collected using the following multiple-choice question: “For which of the following purposes do you use a computer in your practice?” “i) Not applicable (I don’t use a computer); ii) Making appointments; iii) Issuing invoices; iv) Issuing medicine prescriptions; v) Keeping records of consultations; vi) Sending referral letters to medical specialists; vii) Searching medical information on the Internet; viii) Storing diagnostic test results; ix) Sending prescriptions to the pharmacy” (question no. 43, GP questionnaire) [52].

In our survey, e-prescribing was analysed in two operational states of implementation. Generally, the three stages are e-capture, e-transfer and e-dispensation. With answers iv) and ix), we investigated, respectively, the first two states of implementation: the electronic transcription of medication ordering and the electronic sending of prescriptions to pharmacies. Moreover, most of these electronic clinical functions (iv, v, vi, viii, ix) are possible with the adoption of an EHR-like system. Using an electronic record system, other functions can be computerised to manage patient lists, generate a selection from the list and send reminders to patients for preventive care.

The use of computers to communicate with patients was also investigated using the answers to the following question: “How many patient contacts do you have on a normal working day?” (question no. 10, GP questionnaire). This question asked the number of daily face-to-face, telephone and email contacts [52].

Moreover, comparisons over time were performed using data from the TP study, which reviewed the state of PC in 31 European countries and one non-European country in between 1993 and 1994 [53].

With regard to the TP study, coordinated by NIVEL and conducted in 1993, full access to the data was provided by NIVEL. The database includes responses from 7895 GPs from the following countries: Austria, Belgium, Bulgaria, Croatia, Czech Republic, Denmark, Germany, Estonia, Finland, France, Greece, Hungary, Iceland, Ireland, Israel, Italy, Latvia, Lithuania, Luxembourg, the Netherlands, Norway, Poland, Portugal, Romania, Slovenia, Spain, Sweden, Switzerland, Turkey, United Kingdom, Ukraine and Slovakia. The countries in the TP study that were not surveyed in the QUALICOPC study were excluded from our analysis. We used data from the following multiple-choice question in the TP questionnaire: “If a computer is at your disposal, for which purposes is it being used in your practice?” “i) not applicable (no computer); ii) administration/billing etc.; iii) making appointments; iv) recording drug prescriptions; v) keeping patient records; vi) research/audit; vii) other purposes” [53].

*T*-test, chi-square and correlation tests with a 5 % level of significance were used for the analyses described in the following sections. All statistical analyses were conducted using Stata software, version 12.1.

## Results

### Characteristics of the sample

The statistical population for the present study consisted of all GPs in their respective countries for the 31 European surveyed countries (*N* = 6328). Results from the analysis of the sample are reported in Table 2.

**Table 2:** Characteristics of the sample of GPs by country: gender, age, location of practice and typology of practice accommodation (data on 31 European countries from the QUALICOPC survey)

	Characteristics of the sample									
	Sample	Gender	Age	Location of practice			Typology of practice accommodation			Practice population size
	Number	% female	Average	% Big (inner) city	% Suburb/small town	% Urban–rural or rural	Shared accommodation			
							Alone	With other GPs	With medical specialist(s)	Average
Austria	184	30.3	54.3	34.27	23.03	42.70	80.6	8.1	6.1	3096.3
Belgium	408	37.6	49.2	21.38	24.57	54.05	45.4	40.1	2	1494.9
Bulgaria	223	63.2	50.5	49.77	35.48	14.75	63	20.3	9.3	1616.5
Cyprus	71	49.2	48	76.06	19.72	4.23	10.6	64.9	22.3	2384.8
Czech Rep	219	69.9	51.8	27.06	43.12	29.82	82.7	10.4	2.6	1950.3
Denmark	212	43.4	53	26.67	44.29	29.05	21.3	53.7	0	1690.7
Estonia	129	90.5	50.8	43.41	31.01	25.58	65.2	29.6	0.7	2161
Finland	288	71.4	45	16.14	42.11	41.75	27.9	53.6	13	1919
Germany	238	36.1	53.9	23.48	30.87	45.65	48.5	30.4	3.7	2859.5
Greece	220	45.9	43.5	5.91	19.09	75	43.5	38.2	8.8	2457.1
Hungary	222	46.8	53.4	31.22	28.96	39.82	79.3	10.3	3.3	1733.1
Iceland	80	27.5	54.5	37.97	45.57	16.46	1.9	74.3	20.9	1726.9
Ireland	169	33.7	50.6	8.59	37.42	53.99	21.4	57.6	7.1	2768.9
Italy	218	37.6	57.1	25.93	52.31	21.76	43.4	46.3	4.1	1307.8
Latvia	218	88.5	52	41.9	29.05	29.05	84.8	9.2	2.7	1698.1
Lithuania	225	11.5	51.2	85.07	9.05	5.88	29.9	37.1	3.8	1378
Luxembourg	78	36.8	49	14.47	36.84	48.68	38.2	37.2	2.9	3599.6
Malta	70	29	46.7	12.86	58.57	28.57	33.7	43	17.4	3243.8
Netherland	238	28	53	17.09	33.76	49.15	23.6	54.5	3.3	2417.3
Norway	198	39	45.6	33.85	36.41	29.74	0.4	81.3	14.1	1093.4
Poland	220	63.6	49.5	30	36.82	33.18	24.9	50.9	12.6	2420.6
Portugal	216	60.5	51.4	14.42	42.79	42.79	..	..	..	1773.6
Romania	220	83.2	52	33.64	26.27	40.09	34.1	41.5	7.4	1840.3
Slovakia	220	67.9	52.6	18.52	47.22	34.26	93.8	3.1	1.8	1677.4
Slovenia	207	75.4	48.9	35.92	31.55	32.52	71.9	15.3	2.5	1950
Spain	428	63.2	49.7	46.35	36.47	17.18	4	76.9	18.7	1655
Sweden	97	54.6	52	15.46	53.61	30.93	0.9	84.9	6.2	4022.3
Switzerland	199	22.1	55	19.29	28.93	51.78	37.5	37.9	7.9	1678
Turkey	299	30.5	44	73.58	15.72	10.7	10.5	76.9	7.6	3712.3
England	171	37.9	46.6	15.88	44.12	40	5.7	82	5.2	4892.8
FYROM	143	83.9	45.7	54.93	29.58	15.49	34.9	35.4	13.2	1693.8

Source: QUALICOPC

The surveyed countries present various differences in the extent to which GPs play the role of gatekeeper to specialised care. Previous studies have analysed the role of GPs in PC [41]. In general, GPs have a gatekeeping role in systems that are largely public financed but not in those based on social insurance. However, there are countries where the boundaries between general and specialist medicine are blurred, and for this reason, both practitioners and specialists can have the first contact with patients. In countries like Denmark, Iceland, Ireland, Italy, the Netherlands, Norway, Portugal, Slovenia, Spain, Sweden and the United Kingdom, patients go to their GP for referrals to specialists [41]. In other countries, the GP does not have a gatekeeping role. In Eastern Europe, several countries have adopted, but with specific national peculiarities, the Semashko model developed in the former Soviet Union [61], with a greater focus on specialist and hospital care. Some (e.g., Bulgaria, Czech Republic, Estonia, Hungary, Latvia, Lithuania, Poland, Romania, Slovakia and Slovenia) have been transitioning to other models [62], introducing strategic reforms like the gatekeeping role of GPs [63]. Furthermore, countries that score lower in terms of first contact through GPs are in Eastern Europe (Bulgaria, Latvia and Lithuania); however, some (e.g., Hungary and Slovenia) have scores that are equal or higher than those in Central Europe [53].

Several primary health care systems show a gender imbalance among GPs. For example, in the Czech Republic, Estonia, Finland, Latvia, Romania, Slovenia and FYR Macedonia, almost 70 % of GPs are female. In contrast, in Austria, Iceland, Lithuania, Malta, the Netherlands, Switzerland and Turkey, close to 70 % of GPs are male. The mean age of GPs ranges from 43.5 years in Greece to 57.1 years in Italy.

The majority of GPs in Austria, Bulgaria, Czech Republic, Estonia, Hungary, Latvia, Slovakia and Slovenia work alone. In Cyprus, Denmark, Finland, Iceland, Ireland, the Netherlands, Norway, Poland, Spain, Sweden, Turkey and England, GPs share their offices with other GPs. In the remaining countries, there is a mix of the two.

The PCP population estimated by GPs falls generally in the range of 1000–3000 patients (Table 2). Belgium, Bulgaria, Greece, Norway, Switzerland and the former Yugoslav Republic of Macedonia (FYROM) also present an important quota of smaller PC practices (<1000 patients). In contrast, Luxembourg, Malta, Sweden and England have a wide variability in terms of PC practice size, ranging from less than 1000 patients to more than 5000; the average size of Swedish and English practice population is more than 4000 patients per PCP.

The location of the PC practice is another important feature differentiating PC systems. Table 2 shows the distribution of the three location categories (city; suburb or small town; rural) by country. In Belgium and Switzerland, more than 50 % GP practices are located in rural areas. In Cyprus, Turkey, Lithuania and FYROM, there are a greater number of urban PCPs (in Lithuania more than 80 %). Primary health care systems across Europe also vary regarding PCP density. The number of practicing physicians per 1000 persons in the population varies from less than 2.5 in Poland to almost 5 in Austria [64].

The countries also differ regarding GP employment type and remuneration [65, 64].

### Computer use

**Fig. 1:**
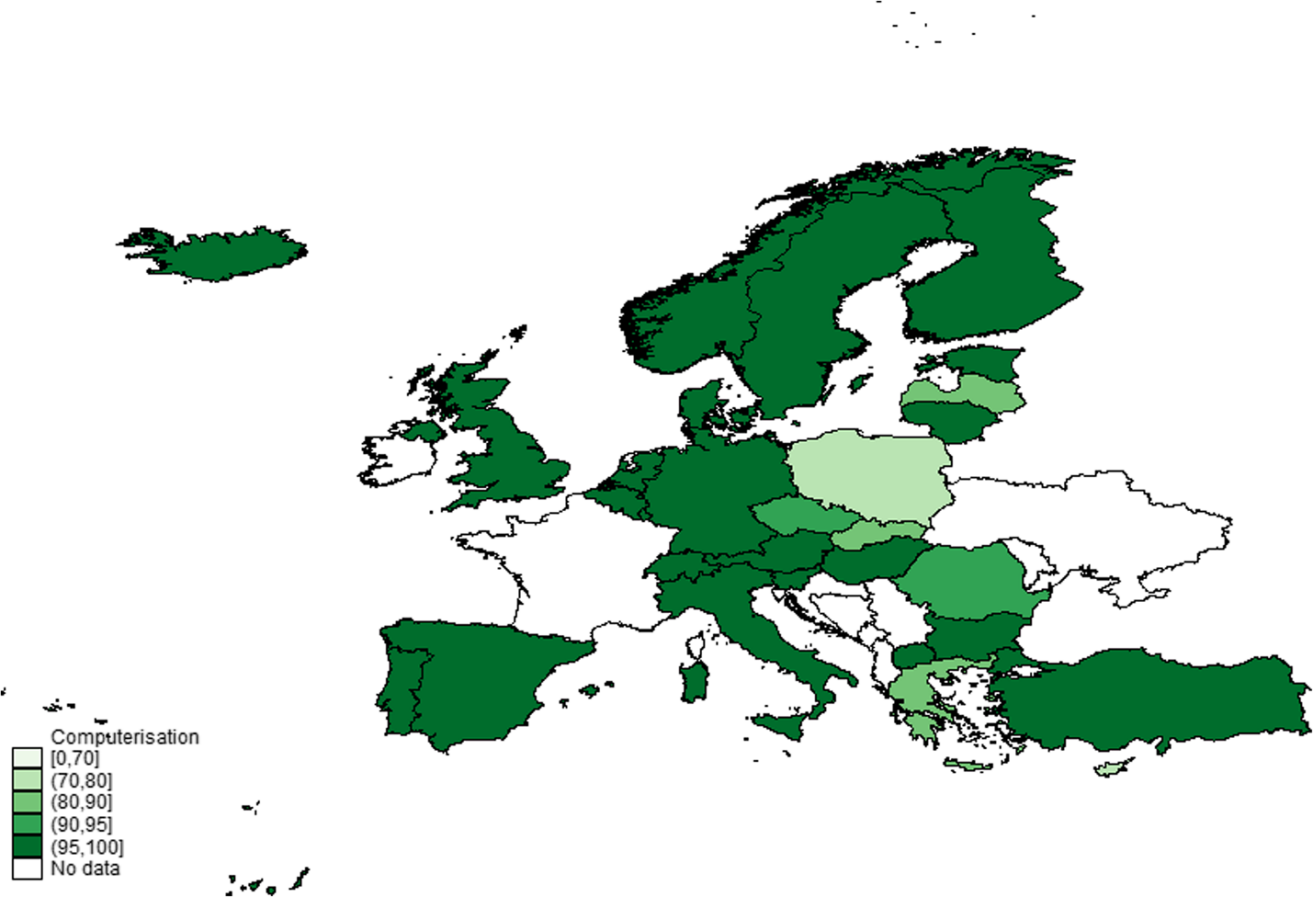
Map of levels of computer use in primary care: comparison among 31 European countries from QUALICOPC survey. The % scales of GPs that reported computer use in their practice range from 70 to 100 %: the first level in the lighter colour represents the range 0–70 %; the second 71–80 %; the third 81–90 %; the fourth 91–95 %; the fifth in the darker colour 96–100 %; the countries in white were not included in the QUALICOPC survey, and for this reason, they have no data and were not assigned a level of computer use in the figure. Base: All GPs. Indicator: question “Computer use”. Source QUALICOPC, 2013

In 2012, almost all the GPs in the surveyed European countries used a computer (96 %) (Fig. 1). Comparing these results with those of the TP study, we found an increase in the use of computers in PC over time: in 1993, computers were used by 40 % of GPs in the surveyed European countries [53].

The results also show a low but significant variability in the general use of computers across and within countries (Fig. 1). The lowest percentage of computer use is in Poland, where 27 % of GPs stated they did not use a computer in their daily PC practice, followed by Cyprus (24 %), Greece (18 %), Slovakia (16 %) and Latvia (13 %). A number of countries reported that 100 % of GPs use a computer in their practice: Denmark, Estonia, Finland, Germany, Hungary, Iceland, Italy, the Netherlands, Norway, Portugal, Spain, Sweden, Switzerland, England and FYROM (Table 3).

**Table 3:** Situation regarding use of computers in primary care in 31 European countries (QUALICOPC survey; indicator question GP43: computer use)

Country	Use of the computer in the PC practice				Cases
	freq	perc	Ci		Freq
Austria	182	99.45	96.99	99.99	183
Belgium	387	95.09	92.51	96.97	407
Bulgaria	219	98.65	96.10	99.72	222
Cyprus	53	75.71	63.99	85.17	70
Czech Republic	206	94.06	90.06	96.80	219
Denmark	212	100	98.28	100^a^	212
Estonia	129	100	97.18	100^a^	129
Finland	285	100	98.71	100^a^	285
Germany	235	100	98.44	100^a^	235
Greece	180	82.19	76.47	87.02	219
Hungary	222	100	98.35	100^a^	222
Iceland	79	100	95.44	100^a^	79
Ireland	164	97.62	94.02	99.35	168
Italy	218	100	98.32	100^a^	218
Latvia	188	87.04	81.81	91.21	216
Lithuania	220	98.21	95.49	99.51	224
Luxembourg	76	97.44	91.04	99.69	78
Malta	67	95.71	87.98	99.11	70
Netherland	235	100	98.44	100^a^	235
Norway	198	100	98.15	100^a^	198
Poland	161	73.18	66.81	78.91	220
Portugal	215	100.00	98.30	100^a^	215
Romania	202	91.82	87.38	95.08	220
Slovakia	182	84.26	78.70	88.85	216
Slovenia	200	96.62	93.16	98.63	207
Spain	428	100	99.14	100^a^	428
Sweden	97	100	96.27	100^a^	97
Switzerland	199	100	98.16	100^a^	199
Turkey	297	99.33	97.60	99.92	299
England	169	100	97.84	100^a^	169
FYROM	142	100	97.44	100^a^	142
Total	6047	95.97			6301

Source: QUALICOPC

^a^one-sided, 97.5 % confidence interval

In some countries, the percentage of practices with computers markedly increased from 1993 to 2012 [41]. Regarding countries with a lower computer adoption rate in 2012, we observe that the percentage of use dramatically increased; for example, from 3 % in 1993 to 73 % in 2012 in Poland, 59 to 82 % in Greece and 34 to 87 % in Latvia. Other examples include Estonia (from 55 % in 1993 to 100 % in 2012), Lithuania (5 to 98 %) and Romania (from 9 to 92 %).

Table 4 summarises the use of computers for all health purposes by GPs across and within countries.

**Table 4:** Use of computers in primary care: comparison among 31 European countries (QUALICOPC survey; indicator question GP43: computer use)

	Making appointments			Issuing invoices			Issuing drug prescr.			Keeping records			Sending ref. letters			Storing test results			Searching med. info			Sending prescript. to phar.			Cases
	Perc.	c.i.		Perc.	c.i.		Perc.	c.i.		Perc.	c.i.		Perc.	c.i.		Perc.	c.i.		Perc.	c.i.		Perc.	c.i.		freq
Austria	58.47	50.97	65.69	81.42	75.02	86.78	93.99	89.50	96.96	96.17	92.28	98.45	96.72	93.00	98.79	92.35	87.50	95.75	83.61	77.43	88.66	15.3	10.41	21.35	183
Belgium	44.72	39.82	49.69	17.20	13.66	21.22	74.45	69.92	78.62	85.01	81.39	88.53	62.9	58.00	67.61	88.94	85.49	91.82	87.47	83.86	90.53	13.02	9.91	16.69	407
Bulgaria	10.36	6.68	15.14	36.49	30.15	43.19	85.59	80.26	89.93	61.26	54.51	67.71	93.24	89.10	96.17	88.74	83.83	92.58	77.93	71.89	83.20	15.77	11.23	21.24	222
Cyprus	8.57	3.21	17.73	7.14	2.36	15.89	1.43	0.04	7.70	14.29	7.07	24.71	12.86	6.05	23.01	75.71	63.99	85.17	45.71	33.74	58.06	2.86	0.35	9.94	70
Czech Rep	39.27	32.76	46.08	76.26	70.06	81.73	87.21	82.05	91.33	84.47	78.99	89.00	65.30	58.59	71.58	62.1	55.32	68.55	84.02	78.48	88.61	9.13	5.67	13.75	219
Denmark	99.06	96.63	99.89	91.04	86.36	94.52	100	98.28	100^a^	99.53	97.40	99.99	99.53	97.40	99.99	99.53	97.40	99.99	98.58	95.92	99.71	99.53	97.40	99.99	212
Estonia	73.64	65.16	81.01	79.84	71.88	86.39	92.25	86.21	96.22	96.12	91.19	98.73	82.17	74.46	88.35	93.80	88.15	97.28	97.67	93.35	99.52	96.90	92.25	99.15	129
Finland	76.49	71.13	81.29	13.33	9.61	17.84	98.60	96.45	99.62	100	98.71	100^a^	98.95	96.95	99.78	97.19	94.54	98.78	96.84	94.09	98.55	38.95	33.25	44.88	285
Germany	55.32	48.72	61.79	93.62	89.69	96.38	99.15	96.96	99.90	87.66	82.76	91.58	99.15	96.96	99.90	88.94	84.21	92.64	71.49	65.26	77.17	16.17	11.70	21.51	235
Greece	4.57	2.21	8.24	0.46	0.01	2.52	75.80	69.57	81.32	26.48	20.77	32.85	24.66	19.10	30.92	26.94	21.19	33.33	58.90	52.08	65.49	9.13	5.67	13.75	219
Hungary	33.78	27.59	40.42	13.06	8.93	18.22	99.55	97.52	99.99	96.85	93.61	98.72	81.98	76.28	86.80	95.50	91.87	97.82	88.29	83.31	92.21	11.71	7.79	16.69	222
Iceland	94.94	87.54	98.60	37.97	27.28	49.59	98.73	93.15	99.97	100	95.44	100^a^	94.94	87.54	98.60	98.73	93.15	99.97	97.47	91.15	99.69	100	95.44	100^a^	79
Ireland	86.31	80.17	91.12	67.26	59.61	74.29	91.67	86.41	95.37	89.29	83.60	93.53	89.88	84.29	93.99	92.86	87.86	96.25	88.69	82.90	93.05	27.98	21.34	35.41	168
Italy	25.23	19.61	31.54	8.26	4.97	12.74	99.08	96.73	99.89	85.32	79.91	89.74	55.5	48.64	62.22	88.53	83.54	92.44	78.90	72.88	84.12	13.3	9.09	18.54	218
Latvia	24.07	18.53	30.34	26.85	21.07	33.29	56.48	49.59	63.19	20.83	15.62	26.87	37.96	31.47	44.80	24.54	18.95	30.83	73.61	67.20	79.36	1.39	0.29	4.01	216
Lithuania	27.23	21.52	33.56	1.79	0.49	4.51	4.46	2.16	8.06	10.71	6.99	15.52	3.13	1.27	6.33	18.75	13.86	24.49	50.45	43.71	57.17	0	0.00	0.02^a^	224
Luxembourg	48.72	37.23	60.31	89.74	80.79	95.47	88.46	79.22	94.59	78.21	67.41	86.76	79.49	68.84	87.80	70.51	59.11	80.30	84.62	74.67	91.79	24.36	15.35	35.40	78
Malta	28.57	18.40	40.62	4.29	0.89	12.02	2.86	0.35	9.94	38.57	27.17	50.97	15.71	8.11	26.38	67.14	54.88	77.91	87.14	76.99	93.95	0	0.00	0.05^a^	70
Netherland	100	98.44	100^a^	94.47	90.73	97.02	100	98.44	100^a^	100	98.44	100^a^	98.72	96.31	99.74	99.57	97.65	99.99	99.15	96.96	99.90	95.32	91.78	97.64	235
Norway	98.99	96.40	99.88	90.40	85.42	94.12	98.48	95.64	99.69	100	98.15	100^a^	99.49	97.22	99.99	98.99	96.40	99.88	97.47	94.21	99.18	90.40	85.42	94.12	198
Poland	25.45	19.84	31.75	27.73	21.92	34.14	50	43.20	56.80	44.09	37.42	50.92	21.36	16.14	27.38	30.45	24.45	37.00	59.09	52.28	65.65	4.09	1.89	7.62	220
Portugal	88.84	83.85	92.72	13.02	8.83	18.27	99.07	96.68	99.89	98.60	95.98	99.71	93.95	89.88	96.74	97.67	94.66	99.24	86.98	81.73	91.17	13.49	9.22	18.79	215
Romania	19.55	14.52	25.41	15.00	10.56	20.42	56.36	49.53	63.02	85	79.58	89.44	32.27	26.14	38.89	32.73	26.57	39.36	76.82	70.67	82.23	12.27	8.25	17.35	220
Slovakia	31.48	25.35	38.13	57.87	50.98	64.54	80.56	74.64	85.61	72.22	65.74	78.08	55.56	48.66	62.30	31.94	25.78	38.61	50	43.14	56.86	5.09	2.57	8.93	216
Slovenia	34.30	27.86	41.20	77.29	70.98	82.81	89.37	84.35	93.22	58.45	51.42	65.24	38.16	31.52	45.15	36.23	29.68	43.18	86.47	81.05	90.82	2.42	0.79	5.55	207
Spain	85.75	82.07	88.92	5.84	3.82	8.50	98.83	97.29	99.62	98.13	96.35	99.19	98.83	97.29	99.62	99.07	97.62	99.74	88.79	85.41	91.61	60.05	55.23	64.72	428
Sweden	95.88	89.78	98.87	23.71	15.66	33.42	80.41	71.11	87.78	96.91	91.23	99.36	95.88	89.78	98.87	95.88	89.78	98.87	98.97	94.39	99.97	100	96.27	100^a^	97
Switzerland	49.75	42.60	56.90	97.99	94.93	99.45	55.28	48.08	62.31	46.23	39.16	53.42	87.94	82.59	92.12	52.26	45.08	59.37	90.45	85.49	94.15	29.15	22.93	35.99	199
Turkey	13.38	9.73	17.77	4.01	2.09	6.91	69.90	64.35	75.05	91.30	87.52	94.24	51.51	45.68	57.30	83.28	78.56	87.33	79.93	74.94	84.32	43.14	37.46	48.97	299
England	98.82	95.79	99.86	60.95	53.16	68.35	99.41	96.75	99.99	99.41	96.75	99.99	98.22	94.90	99.63	98.22	94.90	99.63	98.82	95.79	99.86	44.97	37.32	52.80	169
FYR MO	14.08	8.82	20.91	90.85	84.85	95.03	97.89	93.95	99.56	70.42	62.19	77.78	78.87	71.23	85.27	45.07	36.72	53.64	83.10	75.90	88.86	30.99	23.50	39.28	142
Total	51.33			42.01			80.99			77.40			70.47			73.85			82.18			31.6			6301

Source: QUALICOPC

^a^one-sided, 97.5 % confidence interval

### Use of appropriate ICT applications in primary care

The following computer uses could contribute to an improvement of PC delivery and were analysed: (i) record keeping; (ii) drug prescriptions; (iii) storing of tests results; (iv) making appointments; (v) communication with other parties; and (vi) searching medical information [10]. Some of these computer uses are only based on the availability of a computer, and others on the availability of an Internet connection. The results of the analysis of the different computer uses are reported in Table 4.

In 2012, European GPs mainly used their computers to search for medical information on the Internet and to issue drug prescriptions. In contrast, 1993 data show that the most reported computer use was for “administration” purposes (62 % of GPs) [53].

The use of computers for “searching medical information on the Internet” was reported by 82 % of GPs in Europe. Although this use is widely diffuse across Europe, there are still countries reporting low rates of use. Cyprus has the lowest percentage (46 %), with Slovakia (50 %), Lithuania (50. 5 %), Greece (59 %) and Norway (59 %) also scoring poorly. We found a low but significant variability among countries. This use was less diffuse in 1993, with an average of 37 % of European GPs stating they searched for medical information online. The highest values in 1993 were in the United Kingdom (93 %) and Portugal (77 %), with other European countries presenting values under 50 %. Among countries with lower rates in 2012, it is worth pointing out the marked increase in this use type in Lithuania—in 1993 no GPs used computers to search for medical information.

The second main computer use is “issuing drug prescriptions” (the e-capture stage of e-prescribing). This use was reported by 81 % of GPs and ranged from 1.5 % in Cyprus to 100 % in the Netherlands. We found a huge difference between the 2012 data and the 1993 data. Indeed, the European countries analysed in the TP study presented less than 45 % for computerised prescriptions.

Information systems can also be used to facilitate information sharing and knowledge management with colleagues and specialists. Two computer uses were analysed with this regard: “sending referral letters to specialists” and “sending prescriptions to pharmacies”.

A total of 70.5 % surveyed European GPs use a computer for “sending referral letters to specialists”; in contrast, just 31 % send prescriptions to pharmacies (the e-transfer stage of e-prescribing). Both these uses have high variability among countries: just 3 % of GPs in Lithuania send referral letters to medical specialists via computer compared with 99.5 % in Denmark. This variability also regards sending prescriptions to pharmacies, starting at 0 % in Lithuania and Malta to 100 % in Iceland and Sweden. In these two countries, and in Denmark, Estonia, the Netherlands and Norway, the implementation of a fully operational e-prescribing application appears more advanced. These two computer uses were not analysed in the TP study.

Finally, almost 51 % of European GPs use computers for “making appointments”. Overall, our data analysis showed high variability: the rate varies from 4.5 % in Greece to 100 % in the Netherlands. Compared with the TP study data, we found that in 20 years, the use of computers for this purpose has increased by more than 25 % in Europe [53]. It is also worth highlighting the improvement over time in some countries and the worsening in others. For example, the two countries with the highest and the lowest rates in 2012 had a similar value in 1993: the Netherlands increased from 12 to 100 %, while Greece decreased from 11.5 to 4.5 %.

Denmark, Iceland, the Netherlands, Norway and Sweden emerge as good practices in terms of adoption and use of appropriate ICT applications in PC. In particular, more than 86 % of GPs in Denmark and the Netherlands reported all six appropriate computer uses, while the overall value for all QUALICOPC countries is 59 %. In contrast, we found 10 countries with a rate under the overall average. They can be divided in two groups. The first is composed of countries that range from 40 to 59 %: Romania (39 %), Slovakia (40.8 %), Slovenia (43 %) and Bulgaria (54 %). The second group is composed of countries with values of appropriate computer use under 40 %: Lithuania (14 %), Cyprus (20 %), Greece (28 %), Poland (29 %), and Latvia and Malta (30 %). These countries have considerable room for improvement.

### Storage of data by general practitioners

In terms of patient data storage, two types of computer use were studied: (i) storage of diagnostic test results; and (ii) recording of consultations.

Overall, 75.5 % of GP practices in the 31 European countries stored both types of patient data. In particular, 74 % of GPs stored diagnostic tests results and 77.4 % used the computer for keeping consultation records (Table 4).

The Netherlands, Denmark, Spain, Norway, Iceland, England (99 %), Portugal (98 %) and Finland (97 %) showed the highest percentages of computer use for “storing diagnostic test results”. In contrast, Lithuania (19 %), Latvia (24.5 %) and Greece (27 %) had the lowest percentages.

Regarding the storage of medical records, it seems that the countries can be categorised into four groups. In the first group there are those countries where the rate of GPs using computers for this purpose is lower than 15 %: Lithuania 11 % and Cyprus 14 %. The second group is composed by countries characterised by values ranging from 15 to 49 %: Latvia 21 %, Greece 26.5 %, Malta 38.5 %, Poland 44 % and Switzerland 46 %. The third group presents 11 countries with percentages in the range of 50–89 %: Slovenia 58.5 %, Bulgaria 61 %, FYRMO 70.5 %, Slovakia 72 %, Luxembourg 78 %, Czech Republic 84.5 %, Romania 85 %, Italy 85 %, Belgium 85 %, Germany 87.5 %, and Ireland 89 %. The final 13 countries present the highest rates ranging from 90 to 100 %: Austria, Denmark, Estonia, Finland, Hungary, Iceland, the Netherlands, Norway, Portugal, Spain, Sweden, Turkey and England. Thus, despite a presence of variability among countries, the majority of them widely use computers to store medical records.

The mean value for the European QUALICOPC countries was significantly different from the results of 1993 (almost 18 % higher, *p* < .001) [41].

### Communication with patients: use of email

The computer can be a channel of communication between GPs and patients if there is access to the Internet. The results of the analysis of the different communication channels are reported in Table 5.

**Table 5:** Mean number and type of daily contact between GP and patient: comparison among 31 European countries (QUALICOPC survey/indicator question GP40: daily contacts)

Country	Face-to-face contacts in office		Telephonic contacts		Contacts by email	
	mean	sd	mean	sd	mean	Sd
Austria	48.02	21.40	10.69	10.44	1.65	3.5
Belgium	18.64	7.94	10.40	9.10	0.62	1.76
Bulgaria	32.05	14.75	12.26	10.03	1.36	5.94
Cyprus	29.13	9.77	9.77	5.45	0.26	0.93
Czech Republic	32.87	13.60	11.33	7.5	1.63	2.43
Denmark	23.78	5.59	14.11	7.32	6.30	4.15
Estonia	21.57	8.83	11.76	8.90	2.12	3.11
Finland	12.56	4.85	5.64	3.45	0.34	1.4
Germany	45.5	18.24	9.76	7.00	0.95	5.45
Greece	31.72	14.14	6.81	7.83	0.36	1.47
Hungary	50.43	16.14	11.66	7.94	0.9	2.56
Iceland	13.93	6.88	11.48	5.37	2.86	3.82
Ireland	30.49	10.41	8.90	5.64	0.4	0.88
Italy	27.68	12.25	17.65	11.59	1.86	3.17
Latvia	24.69	7.41	11.63	6.77	1.05	1.76
Lithuania	24.36	7.16	7.61	6.72	0.23	1.05
Luxembourg	25.13	12.63	9.85	7.76	2.27	3.89
Malta	30.17	14.83	13.27	11.85	0.76	2.66
Netherlands	27.86	6.07	8.04	6.50	1.09	3.72
Norway	18.78	4.51	5.86	4.08	1.08	2.78
Poland	34.73	11.91	5.32	5.91	0.39	1.66
Portugal	21.53	6.14	3.18	2.10	0.93	2.17
Romania	25.35	8.21	9.64	7.58	1.33	3.53
Slovakia	46.22	15.35	5.35	4.73	0.54	1.57
Slovenia	45.27	11.31	9.21	6.31	1.52	3.03
Spain	36.22	9.78	3.49	2.65	0.36	1
Sweden	13.03	3.59	6.09	4.33	1.02	2.17
Switzerland	23.96	8.12	6.43	4.31	1.51	2.93
Turkey	61.79	16.47	5.95	6.65	0.2	1.4
England	29.26	6.78	8.95	9.02	0.14	0.5
FYRMO	39.54	14.56	9.65	7.92	1.23	2.96
Total	31.43	16.63	8.7	7.83	1.1	3.01

Source: QUALICOPC

The QUALICOPC survey collected data on the mean number of patient contacts that GPs have on a normal working day, either via face-to-face contact or by phone calls or emails. A total of 34 % GPs reported close to one email communication per day. For all QUALICOPC countries, the number of daily emails was very low (*n* = 1) compared with face-to-face (*n* = 31.5) and telephonic contact (*n* = 8.7).

**Fig. 2:**
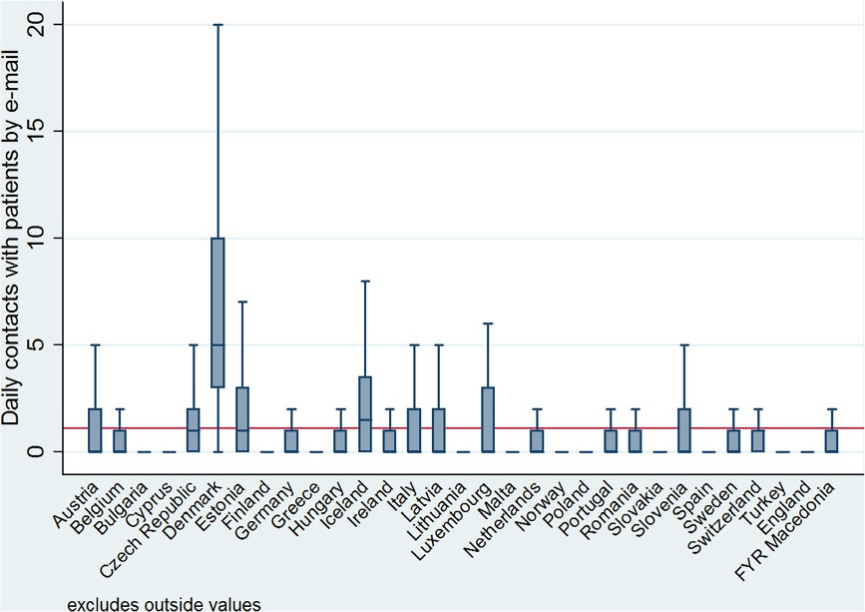
Box plot of the number of daily email contacts per country. Variability among the 31 European countries of the QUALICOPC survey: within countries (dimension of the boxes, position of the median and upper and lower quartiles) and among countries (comparison among boxes). Base: All GPs. Indicator: question “Daily contacts”. Source QUALICOPC, 2013

Looking closer at country-level data, a great variability can be seen (Table 5). In particular, the high variability between countries in the number of daily contacts by email (*p* < .001) is illustrated in Fig. 2: the average number of daily email contacts ranged from 0.13 in England to 6.3 in Denmark. However, it seems that email exchanges with patients were not highly diffused among GPs. Furthermore, 66 % of European GPs have no email contact with patients on a normal working day.

## Discussion

The results of our research have extended the findings of the TP study [53] by documenting the extent to which PC physicians use ICT to provide patient care in 31 European countries in 2012.

The first finding of our study is that the computerisation of general practices has grown over the past two decades. A near-universal adoption of computers in PC has been achieved by all the sample countries. Only five countries show adoption rates below 90 %: Poland, Cyprus, Greece, Slovakia and Latvia. These findings confirm the results of another recent survey [51].

As revealed in previous studies [30, 51], our results show that although computers are considered PC equipment, their use for clinical purposes is significantly different across Europe. North European countries (Denmark, Iceland, the Netherlands, Norway and Sweden) have the best results in terms of appropriate use of ICT in PC. In contrast, several Eastern (Czech Republic, Lithuania, Poland, Slovakia and Slovenia) and Southern European countries (Cyprus, Greece, Malta and Turkey) present a lower use of basic ICT, both in 2012 and in 1993. However comparing QUALICOPC and TP data, we found that these countries have had a growth in computer adoption and use in PC in the last 20 years. In particular, ICT infrastructures and use in PC systems of Estonia and Hungary has improved over time, achieving good results if compared with other European countries.

Several interesting explanations to the geographic variability in the ICT adoption have been found by analysing selected variables related to PC context.

Similar to previous research [30, 33–38], our results show that, in general, the size of the practice is associated with ICT use: in single-GP practices the computer is used less than in offices shared with other GPs or specialists. This association is significant for the storage of test results (ρ = −.41, *p* = .02) and for sending prescriptions to pharmacies (ρ = −.56, *p* = .001). Although these two uses has not achieved universal implementation, they seem to be favoured by an inter-professional or networked PC.

EHR-like systems and e-prescription are priorities in various EU e-Health Action Plan and in the policies of several Member States (respectively, 27 and 22 EU countries) [31, 66]. However, the general political commitment to these e-health fields is at different stages of implementation across countries [31]. While, e-capture is a widespread activity (reported by 81 % of GPs), with a significant variability among countries, the use of e-transfers by GPs is low (31 %). We only found a near-complete operational e-prescribing application in just a few Northern European countries, including Estonia. The low use rate of a connected computer to collaborate with other parties might be explained by privacy and data security concerns, in addition to other contextual factors such as cultural barriers (i.e., perception of low usefulness of this typology of ICT applications) and technological or infrastructural factors (i.e., availability or interoperability of health records or e-prescriptions systems).

In contrast, the high diffusion (82 %) and low variability across Europe of the computer use to search for online medical information might suggest: (i) an increased trust and awareness of GPs upon the quantity and quality of available knowledge on the Internet; (ii) its use as mainly personal support in daily practice, not for collaboration with other physicians.

Although the e-health policies at European and national levels also recognise the potential benefits of electronic records as a key tool for continuity and quality of care, this electronic function has not achieved universal implementation (77.4 % of the surveyed GPs use electronic records, with a large variability between countries). These results could be explained, again, by various factors at the country level, such as interoperability problems, safety and privacy issues and the centralisation or delocalisation of data control. In fact, standardisation and legal and regulatory issues are among the key applications of European e-health strategies [67].

No statistically significant results were found for the association between practice location (rural) and computer use. Whereas, considering the number of elderly patients in the PCP population, we found that the presence of elderly patients below the country average is negatively associated with some computer uses in PC (ρ = −.43, *p* = .01). These results might be explained by the fact that aged patients are the highest users of healthcare services, especially PC services, and also the most fragile patients owing to chronicity and multi-morbidity [37].

Looking at country level, variations across European countries could be partially explained by national policies on e-health, as anticipated. All European countries have adopted policies on e-health, or participated in European projects and discussions [31, 68]. Central and Northern European countries (e.g., Scandinavian countries, Germany and Denmark) enjoy mature e-health strategies. In contrast, Southern and Eastern EU countries are mostly at the planning stage or only now implementing e-health applications. Among Eastern European countries, Estonia represents a positive example also in terms of strategic e-health applications [63].

Referring to World Information Technology and Services Alliance data [69] adjusted at current purchasing power parities (PPPs) [64] we did not find a significant association between ICT expenditure in the health care sector and ICT adoption and use in PC. This might be explained by the fact that computers have been widely adopted in all QUALICOPC countries and do not require large financial investment. Therefore, expenditure on ICT might have a significant impact on advanced e-health applications.

Similar results were found by analysing 2012 OECD data on the total expenditure on health care per capita [67] adjusted at current PPPs [64].

It seems that some variations can be explained by the size of the public resources spent on health care. Significant positive associations (ρ = .05) were found between the public expenditure on health care and both the computerisation rate (*p* = .04) and several computer uses (*p* = .05). By analysing our results in association with health system typologies, we also found negative significant associations between ICT use and countries with a system in transition from the Semashko model (ρ = −.45, *p* = .05).

Another finding of this study is that doctor–patient communication via email is not a common practice. Face-to-face communication appears widely preferred to other forms of contact with GPs. We found a very low rate and variability regarding email communication. Denmark is the exception with a mean value of 6.3 contacts per day. While Denmark is a leading country in the use of health care technology, these results can be better explained by the innovations introduced in the Danish health care system, including payments to physicians for email with designated call-in times [70].

## Conclusions

The findings of the present study reveal significant progress in basic ICT adoption in European PC. Several countries have achieved a universal adoption of computer use in just a few years, and other countries are following closely behind. Significant differences in the uses of computer as reported by GPs were found across and within countries. These variations might be partly explained by differences in the organisation of health care systems, the role of GPs, funding models, national and local policies and legislation on e-health.

The requirement for appropriate technological innovation in PC is growing, and is now seen as an important contribution to the improvement of quality and efficiency in health care. However, the variability and/or low use of computerised PC functions reveal that having a computer in a physician’s office is not sufficient.

In addition, the increasing availability of ICT solutions from external PC networks, such as laboratories and pharmacies, as well as the increase of expert patients, and their growing recourse to the Internet for health purposes [19, 71–75], are changing the context in which physicians operate. GPs must become increasingly aware of the opportunities provided by technological innovations in PC. In this sense, evidence-based education on these topics for medical staff is crucial. Organisational support in terms of culture and environment is also fundamental.

An appropriate and comprehensive use of ICT in PC could be incentivised by specific policies aimed at building a connected and collaborative health care system. The introduction of ICT in health care could change work tasks and boundaries. Public policies may be based on a general agreement of health care workers on the processes of technology adoption.

Technological innovation concerns not only technologies, but also behavioural, organisational and networking aspects, becoming a disruptive innovation [1]. For this reason, the results of this research emphasise the crucial role in a successful innovation process of public authorities, at European, national and regional levels.

## Abbreviations

GP(s): General practitioner(s)
ICT: Information and communication technologies
QUALICOPC: Quality and costs of primary care in Europe
PC: Primary care
EU: European union
EU-27: European union of 27 member states (until 30 June 2013)
EHR(s): Electronic health record(s)
FYROM: Former Yugoslav Republic of Macedonia
TP: Task profiles
SSSA: Scuola Superiore Sant’Anna
